# Influence of Methodological Variables on Fracture Strength Tests Results of Premolars with Different Number of Residual Walls. A Systematic Review with Meta-Analysis

**DOI:** 10.3390/dj9120146

**Published:** 2021-12-02

**Authors:** Carlo Gaeta, Crystal Marruganti, Emanuele Mignosa, Giovanni Franciosi, Edoardo Ferrari, Simone Grandini

**Affiliations:** Unit of Endodontics and Restorative Dentistry, Department of Medical Biotechnologies, University of Siena, 53100 Siena, Italy; marruganti@gmail.com (C.M.); emanuele-mignosa@outlook.it (E.M.); giofrancio@libero.it (G.F.); edoardo.ferrari.cagidiaco@gmail.com (E.F.); grandini@unisi.it (S.G.)

**Keywords:** fracture strength test, endodontically treated teeth, premolar

## Abstract

The aim of the current meta-analysis was to assess the impact of methodological variables in performing fracture strength tests of upper premolars. Medline (Pubmed), Embase and Google Scholar were screened for studies performing ex vivo fracture strength tests of intact upper premolars or premolars with 0, 1 or 2 walls lost. The outcome variable for each study was the maximum breaking load expressed in Newton (kg × m/s^2^). Methodological variables (i.e., simulation of the periodontal ligament, load inclination, tip position, tip diameter and thermocycling) were registered to perform subgroup analyses and meta-regression. Overall, 25 studies and 78 study groups were included in the meta-analysis. Intact premolars (17 study groups) were not significantly influenced by any of the methodological variables considered. Subgroup analysis for load inclination (30°/45° vs. 90°/150°) was significant for premolars with 0 (10 study groups), 1 (6 study groups) and 2 (45 study groups) walls lost; thermocycling was significant for premolars with 1 and 2 walls lost. A strong methodological heterogeneity across studies evaluating the fracture strength of upper premolars was highlighted, especially when 0, 1 or 2 walls were lost. Further studies are needed to standardize the methodology used in order to allow for across-studies comparisons.

## 1. Introduction

Restorative procedures and Endodontic treatment produce extensive loss of dentine structure favoring risk of fracture and tooth loss [1,2]. It is widely accepted that the extension of access cavity during endodontic treatment and consequently the number of walls lost as well as the removal of occlusal marginal ridges could sensibly affect the strength of the teeth involved [3]. Upper premolars are more prone to fracture compared to molars because of their position in the mouth and the anatomical features, such as the shape, crown volume and crown/root proportion [4]. Fracture strength test remains a common experimental method to evaluate the restorative procedure for root filled teeth despite shortcomings being highlighted regarding the correct physiological load, the teeth used in the experiment, and the differences in test conditions [5]. The pattern of loading plays a crucial role in the fracture strength test, in which it tends to simulate the occlusal forces in the mouth. The direction and location of the loading tip as well as the shape and the diameter may influence the results of the test. Usually, the direction of the applied forces, used in this in vitro test, are axial on both buccal and palatal cusps with an inclination of 30/45°, and this could sensibly influence the outcome of the test [6]. The periodontal ligament is an anatomical structure able to distribute the occlusal load thanks to its fibers. Its inclusion in the fracture strength test is confirmed by a finite element analysis study [7] and it seems to influence the results of the in vitro test [8]. Thermo cycling is an in vitro procedure used to simulate the thermal stresses that usually occur during masticatory function [9]. This tooth aging treatment seems to considerably affect the results, if applied [10]. To date, no systematic review or meta-analysis is present regarding the impact of methodological variables related to the experiment on the fracture strength of the teeth evaluated. Such results may have an implication regarding the testing phase of different restorative materials, and could also serve as a guide for future studies. The aim of the present systematic review is to evaluate the influence of methodological variables such as Thermocycling, Periodontal ligament simulation, Load inclination and Tip diameter, on fracture strength test of upper premolar with intact, 0, 1, 2 walls lost.

## 2. Materials and Methods

### 2.1. Protocol and Registration

The present systematic review was performed following the PRISMA (Preferred Reporting Items for Systematic Review and Meta-Analyses) statement [11].

### 2.2. Eligibility Criteria

All ex vivo studies performing a fracture strength test on premolars, extracted for orthodontic and periodontal reasons were included in the present protocol. Inclusion criteria were defined with the PICO(S) method:

- Population: intact premolars or with 0/1/2 walls lost (i.e., intact premolars, or premolars without the involvement of any of the 4 walls, or premolars with either the mesial or distal wall lost, or premolars with both the mesial and distal walls lost);
- Intervention: fracture strength test;
- Comparison: no comparison group was defined;
- Outcome: fracture strength, measured in Newton;
- Studies: all ex vivo studies.

#### Exclusion Criteria

All studies where an indirect restoration, fiber post or endocrown were performed, were excluded from the current protocol.

### 2.3. Information Sources

**Figure 1:**
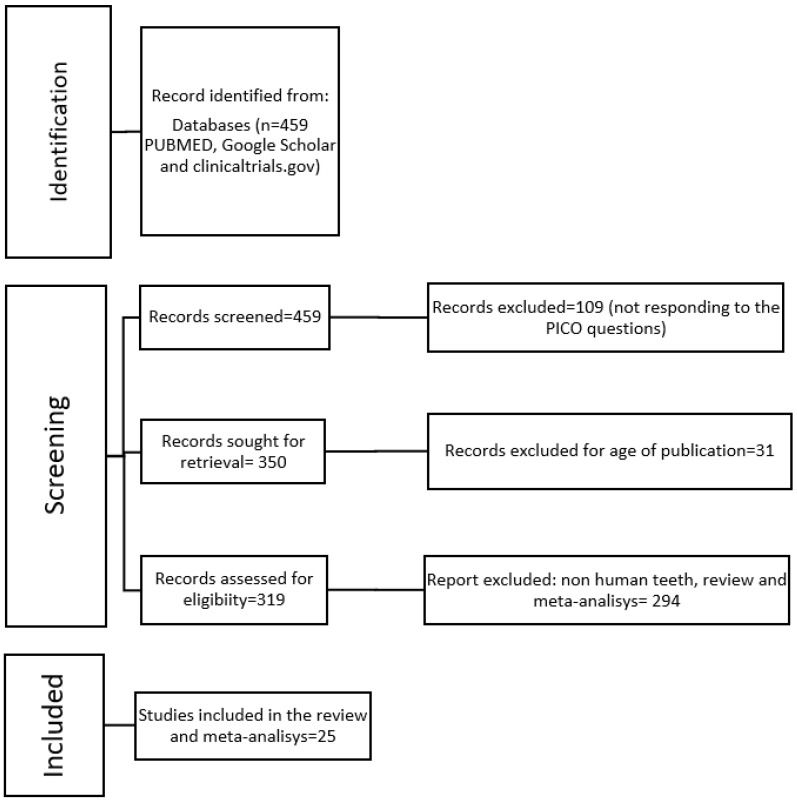
PRISMA flow diagram summarizing all inclusion criteria.

The search strategy included the screening of electronic databases and the relevant journals. Other sources encompassed citations from relevant articles. The screening and inclusion stages were reported following the PRISMA flow diagram (Figure 1).

#### 2.3.1. Electronic Search

The electronic databases screened were MEDLINE (through Pubmed) and Google Scholar; a search for grey literature was also carried out (OpenGrey). A combination of MeSH terms and free text words was used to define the full electronic strategy. Only studies published in English or Italian were included; no restrictions as to publication date were applied.

The full electronic search strategy is reported for MEDLINE (through PubMed):

Fracture Strength Test AND (endodontics OR endodontically treated teeth).
The search strategy was then adapted for the other databases.

#### 2.3.2. Hand Search

Relevant journals (*Journal of Endodontics*, *International Journal of Endodontics*, *Journal of Prosthetic Dentistry*) were screened by two independent reviewers (C.M., C.G.), assessing all issues from January 1985 until June 2021. Other sources encompass narrative reviews and references from relevant articles.

### 2.4. Study Selection

Titles and abstracts were independently screened for relevance by two calibrated reviewers (un-weighted Cohen’s *k* score of 0.90) (C.M., C.G.). Subsequently, the pre-selected articles were screened for full-text analysis by both reviewers according to the eligibility criteria. Any disagreement at any stage (title/abstract or full text) was resolved through discussion with a third author (S.G.) in order to reach consensus.

### 2.5. Data Collection Process

Data collection was performed through an extraction sheet by two independent reviewers (C.M., C.G.) during full-text analysis. Characteristics of the included studies encompassed: fracture strength test of intact upper premolars or with, 0, 1 and 2 walls lost.

### 2.6. Data Items

The primary outcome of the protocol was defined as the maximum fracture strength value expressed in Newton (kg × m/s^2^). The methodological variables such as Periodontal Ligament (PDL) simulation, inclination of the load, tip position and diameter, thermocycling were registered and used to perform subgroup analysis and meta-regression.

### 2.7. Summary Measures

In the present systematic review, fracture strength values were considered as the main outcome and a precomputed effect size with 95% Confidence Interval was calculated. Whenever it was not available, fracture strength was calculated through raw data analysis.

### 2.8. Synthesis of Results and Additional Analyses

Statistical analysis was carried out through an ad hoc statistical software (STATA BE, version 17, StataCorp LP, College Station, TX, USA).

Cochran’s Q statistic and the *I^2^* index were used in order to estimate heterogeneity across studies. Between-study variance was estimated with the T^2^ parameter. Meta-analysis of the included studies was conducted through an inverse variance analysis using the Der Simonian and Laird random effects model. The analysis was performed using the pre-computed effect size pooled for each study. Subgroup analysis was performed (for each group: intact premolars, 0, 1, 2 walls lost) about the following variables:

Periodontal ligament simulation;
Load inclination;
Tip position;
Thermocycling;

Meta-regression of the covariate Tip diameter was also performed. The obtained results were graphically showed by forest plot. Moreover, publication bias was assessed through the Egger’s test. Values of *p* < 0.05 were considered statistically significant.

## 3. Results

### 3.1. Study Selection

Initial electronic search retrieved a total of 459 articles in MEDLINE. Manual search in relevant journals led to no additional records. 31 were excluded because for age of publication. After the exclusion of 294 records at the eligibility level, 134 were assessed for full-text eligibility. Finally, 25 studies were included in both the qualitative and quantitative (meta-analysis) synthesis of data, from which 17 subgroups were extrapolated for Intact premolar, 17, 6, 45 respectively for 0, 1, 2 walls lost. Inter-examiner agreement for articles inclusion resulted in a Cohen’s *k*-score of 0.90. The study selection process is depicted in Figure 1. Characteristics of the included studies are described in Table 1.

### 3.2. Descriptive Analysis

Intact premolars

Out of 17 subgroups, the load inclination applied was 90° for nine [10,12,13,14,15,16,17,18,19] and 30°/45° for eight [19,20,21,22,23,24]. PDL simulation was performed in five subgroups [10,12,15,22,25] while thermocycling was applied in five [10,12,15,23,26]. Tip diameter was respectively lower than 2 mm and between 2 and 3 mm in one group, between 3 and 5 mm in four groups, 6 mm in seven groups and 8 mm in two groups.

Premolar with 0 wall lost

Out of 17 subgroups, the load inclination applied was 90° for two [27] and 45° for six [17,19,28]. Thermocycling simulation was performed in two subgroups [17,29] while no PDL simulation was applied. Tip diameter was respectively 3 mm in three groups and 6 in seven.

Premolar with 1 wall lost

Out of 6 subgroups, the load inclination applied was 90° for four [14,28,30] and 45° for two [14]. Thermocycling was performed in one subgroup [14] while no PDL simulation was applied. Tip diameter used was between 2 and 3 mm in one group while in the remaining was not specified.

Premolar with 2 walls lost

Out of 45 subgroups, the load inclination applied was 90° for twenty-four [10,12,22,30,31,32,33,34] and 30°/45° for twenty-one [10,13,15,19,23,28,35,36]. PDL simulation was performed in twelve subgroups [10,15,22,30,31] while thermocycling was applied in six [10,30,31]. Tip diameter was respectively 3 mm in three groups and 6 in seven. Tip diameter was respectively lower than 2 mm in nine and between 2 and 3 mm in one group: between 3 and 5 mm in four groups, 6 mm in thirteen groups and 8 mm in seven. In remaining groups, the tip diameter was not specified.

### 3.3. Meta-Analysis

Intact premolars

**Figure 2:**
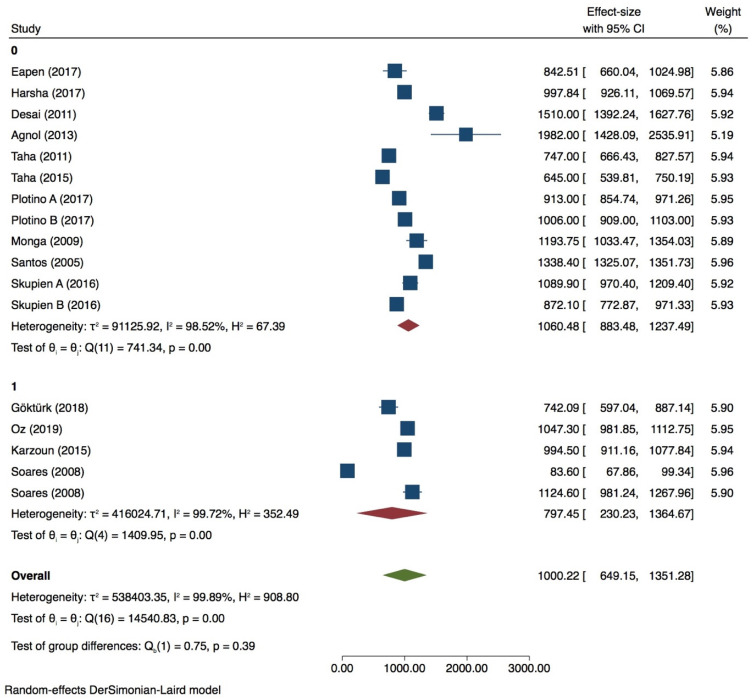
Forest plots for intact premolars of PDL simulation.

**Figure 3:**
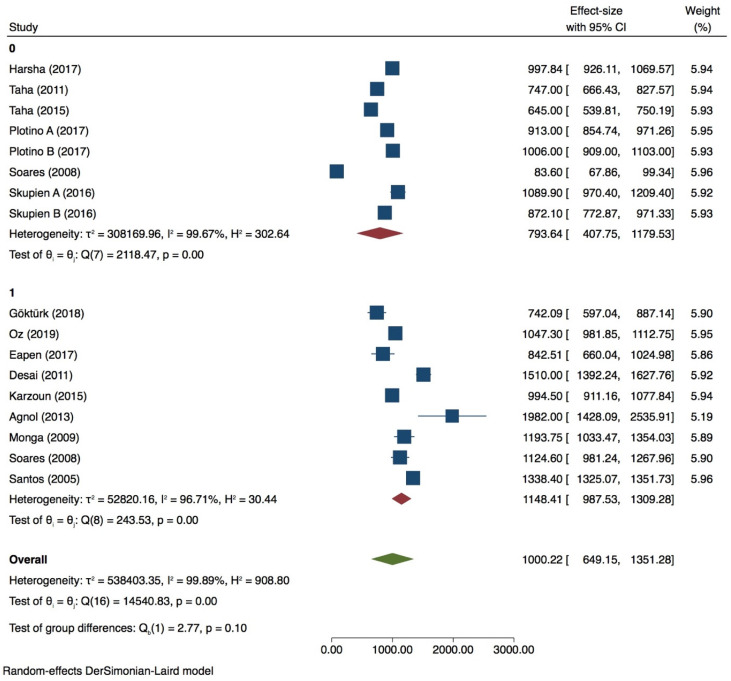
Forest plot for intact premolars in load inclination subgroup.

**Figure 4:**
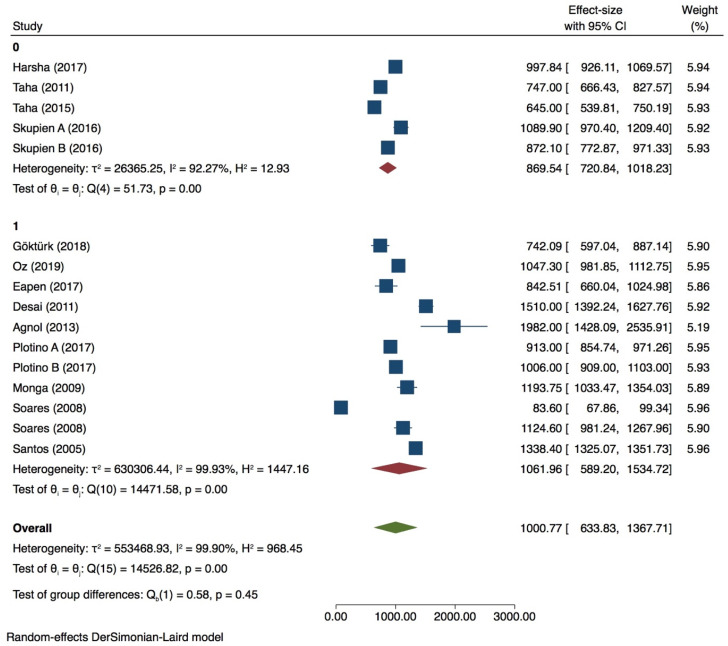
Forest plot for intact premolars in tip position subgroup.

**Figure 5:**
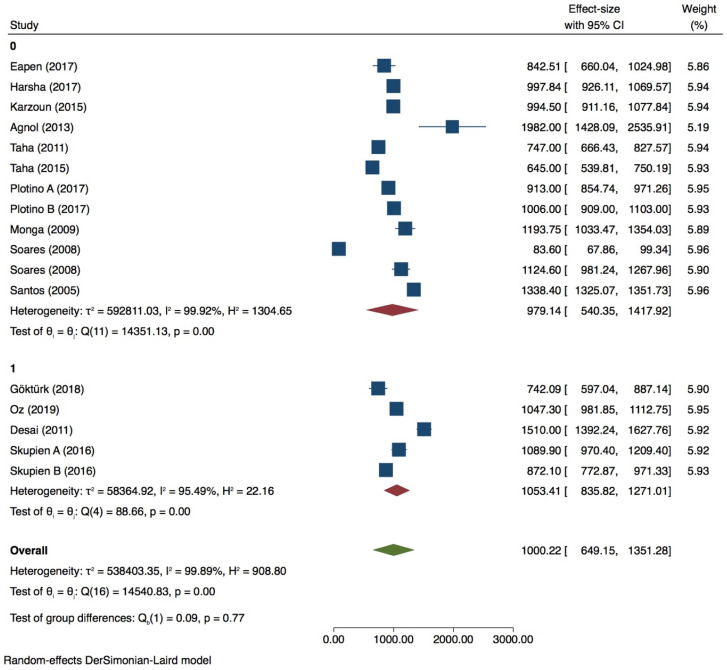
Forest plot of intact premolars in thermocycling subgroup.

Thirteen groups were included for meta-analysis with very high heterogeneity value (*I^2^* = 99.90%) (Figure 2, Figure 3, Figure 4 and Figure 5) while publication bias was not present (*p* > 0.05) (Supplementary Materials, Figure S1). Subgroup analysis showed that none of the variables analyzed, influences the fracture strength value in intact premolars (*p* > 0.05) (Table 2). Meta-regression of the tip diameter did not influence the fracture strength of the intact premolar.

Premolar with 0 wall lost

**Figure 6:**
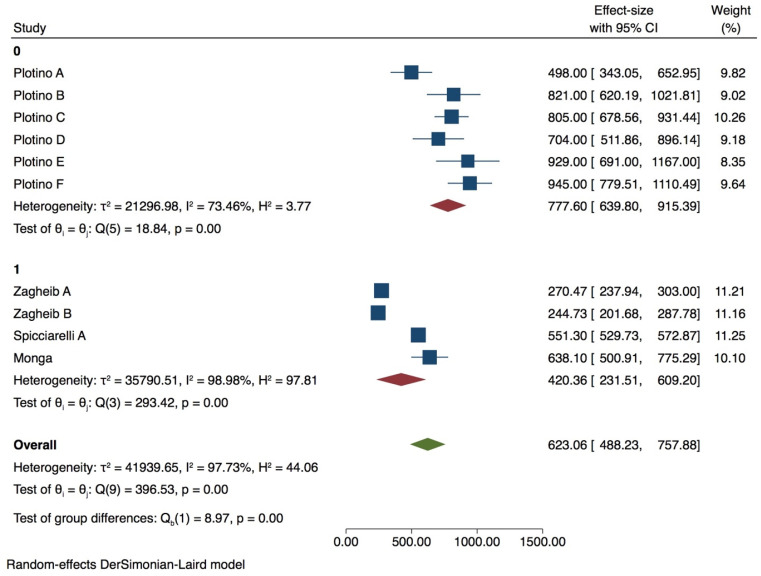
Forest plot of premolar with 0 wall loss of tip inclination variable.

A meta-analysis was performed with 10 groups. The Heterogeneity was very high (*I^2^* = 97.73%) (Figure 6), and the publication bias was present (*p* = 0.00) (Supplementary Materials, Figure S2). PDL simulation significantly influences the fracture strength test (*p* = 0.00), as well as the 90° tip inclination when compared with 30/45° (*p* = 0.00) (Table 3). Tip position and thermocycling does not affect the fracture strength test of premolars with 0 wall lost (*p* > 0.05). Meta-regression of tip diameter does not seem to affect the test (*p* > 0.05).

Premolar with 1 wall lost

**Figure 7:**
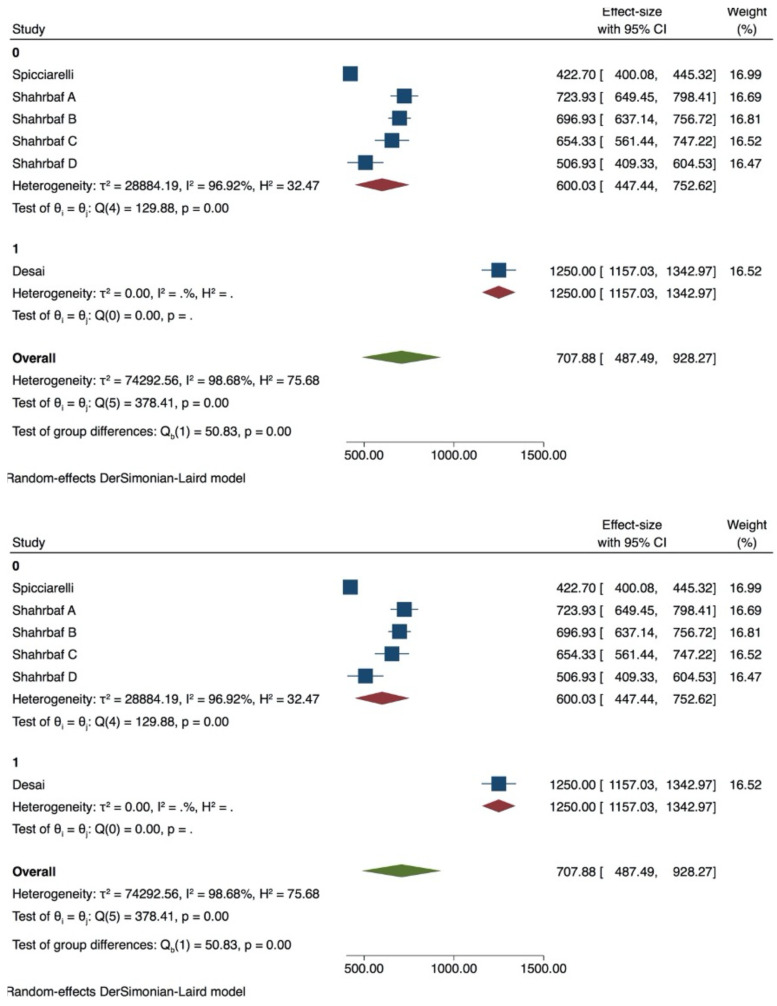
Forest plots of thermocycling and tip position in premolar with 1 wall lost.

6 groups were included in the meta-analysis. Despite a high heterogeneity (*I ^2^* = 97.73%) (Figure 4), publication bias was not present (*p* > 0.05) as shown also by the funnel plot (Supplementary Materials, Figure S3). Tip position and thermocycling significantly influence the test on premolar with 1 wall lost (*p* = 0.00) (Figure 7), while inclination did not (*p* > 0.05) (Table 4).

Premolar with 2 walls lost

**Figure 8:**
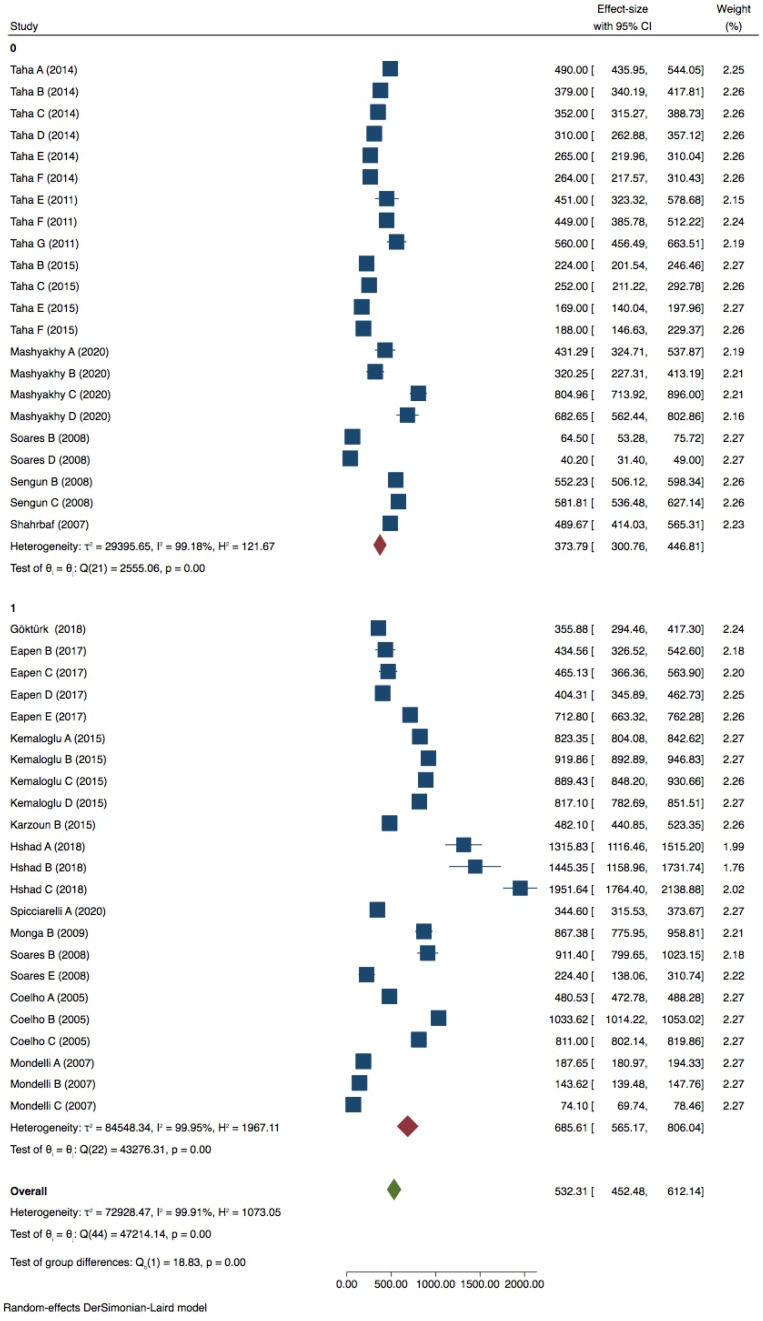
Forest plot of in premolar with 2 walls lost group.

**Figure 9:**
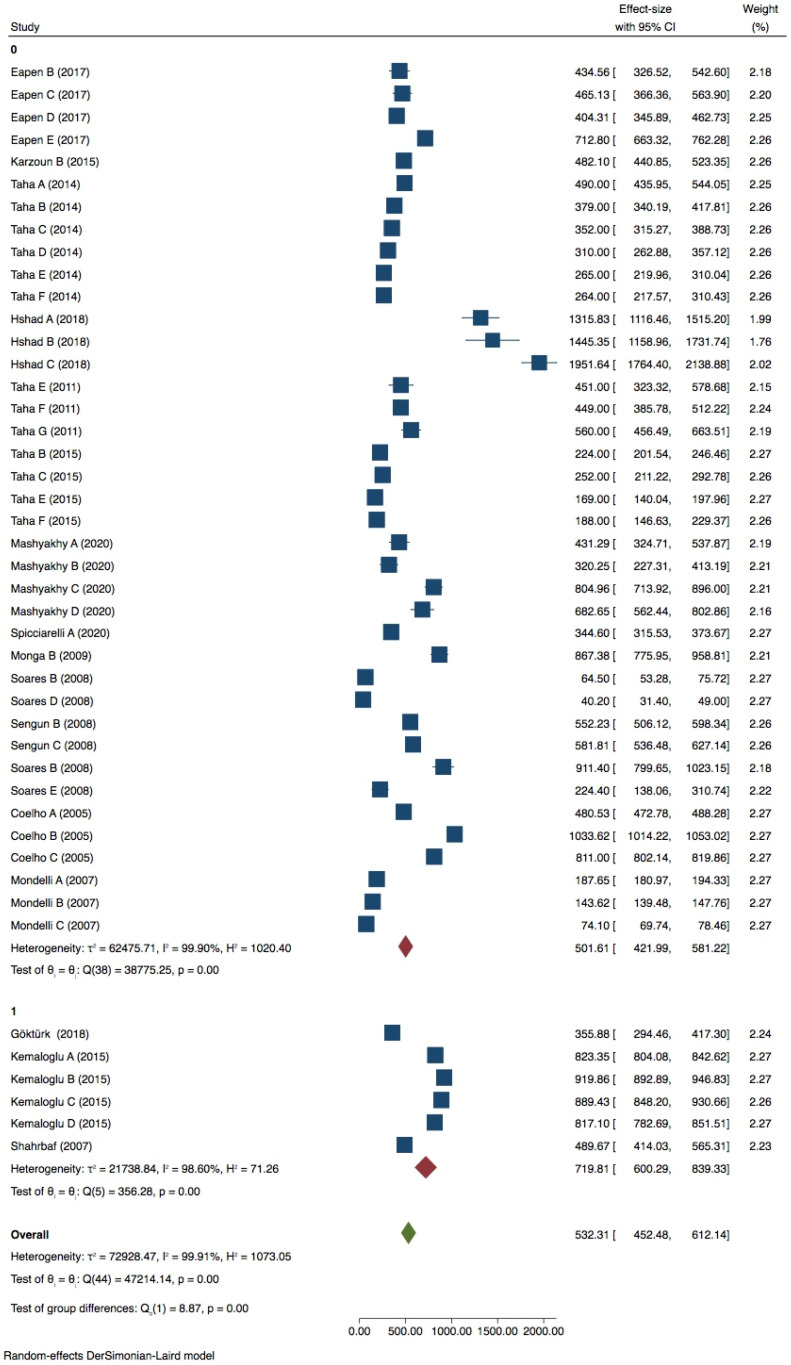
Forest plot of tip inclination in premolar with 2 walls lost.

A meta-analysis conducted with 45 groups showed a very high heterogeneity (*I^2^* = 99.91%) (Figure 8), and the Egger test indicated the presence of publication bias (*p* < 0.05) (Supplementary Materials, Figure S4). PDL simulation showed no influence on the fracture strength test (*p* > 0.05). 90° tip inclination significantly affected the test compared to 30/45° (*p* = 0.05) (Figure 9). Neither the tip position nor the diameter seemed to influence the results of the in vitro test (*p* > 0.05) (Table 5).

## 4. Discussion

### 4.1. Summary of Findings

In the present meta-analysis, we evaluated the impact of methodological variables such as periodontal ligament simulation, tip diameter, position and inclination, thermo cycling in the fracture strength test on the upper premolar with different residual walls. Studies regarding premolars were chosen because of their natural tendency to be more prone to fracture compared to other teeth, although anatomical variables such as crown volume and inclination of the cusp could affect the strength of the tooth. According to the results of the present study, all methodological variables, including periodontal ligament simulation, thermocycling, tip position and diameter, load inclination, do not seem to affect fracture strength test in the intact premolar, whereas a very significant publication bias and high heterogeneity were present in this subgroup. In this group the fracture strength does not seem to be affected by the variables analyzed, showing, as expected, the higher value of fracture strength compared to other groups as confirmed previously by da Angol et al. [20] and Jantarat et al. [37]. This could be explained by the homogeneous distribution of load force transferred from the rigid intact enamel to the underlying dentin as demonstrated by Ausiello [38]. Periodontal ligament simulation influences the fracture strength test only in the premolar with 0 wall lost in respect to the other three groups examined in this review. Periodontal ligament represents an important anatomical structure able to absorb the occlusal forces during chewing function and should be reproduced in the “in vitro test” in order to simulate the clinical reality. Rees [8] underlined the importance of simulating the periodontal ligament in the fracture strength test by a finite element analysis. In any case, many materials were utilized to perform these procedures and a lack of uniformity between studies was observed in the review analysis.

Metaregression regarding the variable tip diameter seemed to not influence the test in any case. In addition, in this case, new studies are needed to understand if the diameter of the tip could influence the fracture strength test. The load inclination seemed to influence the fracture strength test when the force was applied at 30/45° instead of perpendicular, except for premolars with 0 wall lost. Yang [39] showed that the direction of the force decreases the fracture strength as the walls lost increase. The same results were obtained by Reeh [8] in case of endodontically treated teeth, demonstrating a higher resistance to fracture when conservative cavity access was applied. According to our data, thermocycling seems to influence with statistical significance the data of the fracture strength test mostly in the case of premolars with 1 and 2 walls lost, showing higher resistance to fracture when the procedure was applied. Our results are in agreement with Sabery [40] regarding intact premolars, whereas his showed that thermocycling influenced the fracture strength values in the case of different cavity preparation. These data could be explained by the fact that thermocycling is applied to put in evidence teeth with crack or fracture already present which could influence the results of the test.

### 4.2. Strengths and Limitations

The current review represents the first meta-analytical analysis of the methodological variables present in the fracture strength and their influence on the test results. The strict methodology used according with new PRISMA guidelines, the high number of the included study as well as the high number of the methodological variables analyzed represent the strength of the present study. Despite the lack of publication bias, outcome reporting bias could be present, affecting the heterogeneity of the selected studies. In fact, despite additional analysis, it was impossible to reduce the heterogeneity, thus suggesting that other methodological variables, as well as ignoring non-significant outcomes, which could not be considered, may have influenced the results. The present meta-analysis showed high heterogeneity values in all groups analyzed and this represents the main limitation of the present study in which the results should be considered with caution.

Data were scarce about periodontal ligament simulation, tip position and diameter and in any case, our meta-analysis fails to detect a statistical significance in the fracture strength test, despite it having been observed that a decrease of the tip diameter matches a lower resistance to fracture [37]. Another limitation of this systematic review is represented by the lack of information regarding the choice of what upper premolar has been used. Taha et al. [5] reported the influence of shape difference of cervical area between first and second premolar in the fracture susceptibility.

## 5. Conclusions

Fracture strength test is the main in vitro study able to better understand the capacity of dental materials to resist under stress conditions and in various clinical situations. Despite the numerous studies already published in the literature, there is an evident lack of uniformity. The present meta-analysis highlights the necessity to standardize the procedure in order to reduce the variability of fracture strength test results.

## Figures and Tables

**Table 1:** characteristics of the included studies.

Authors	Years	Teeth	N	PDL	Thermocycling	Load Inclination	Tip Application
Gokturk	2018	Upper	55	+	+	90°	Buccal/Lingual cusp
Oz	2019	Upper	80	+	+	90°	Occlusal surface
Eapen	2017	Upper	60	-	--	90°	Occlusal inclines of the Buccal and Lingual cusps
Harsha	2017	Upper 1 st	40	-	+	30°	On the Center of the Buccal cusp
Desai	2011	Upper1 st	30	-	+	90°	Center occlusal surface
Kemaloglu	2015	Lower	48	+	-	90°	B and L cusps simultaneously
Zogheib	2018	Upper	60	+	-	90°	Central fossa
Karzoun	2015	Upper	60	+	-	90°	/
Taha	2014	Upper	48	-	-	45°	/
Hshad	2018	Lower	48	-	-	90°	Interdental surface of the buccal cusp
Angol	2013	Upper	10	-	-	90°	Simultaneous contact B and P cuspal inclines
Taha	2011	Upper	80	-	-	45°	Palatal incline of Buccal cusp
Taha	2015	Upper	77	-	-	45°	Palatal incline of Buccal cusp
Mashyakhy	2020	/	52	+	+	90°	Center occlusal surface
Spicciarelli	2020	Upper 1st (1 root)	165	-	-	90°	2 mm from apex of Palatal cusp in the direction of Central fossa
Monga	2009	Upper	80	-	-	90°	Occlusal inclines of the buccal and lingual cusps
Soares	2008	/	50	+	-	45°	Buccal and lingual cusps
Sengun	2008	Lower	80	+	-	45°	Central fossa with lingual orientation
Soares	2008	Upper (1 root)	50	+	-	90°	Center occlusal surface
Shahrbaf	2007	Upper	60	-	+	45°	Palatal cusp
Coelho	2005	Upper	90	-	-	90°	Buccal and Lingual inclined cuspal planes, not restoration
Mondelli	2007	Upper 1st	40	-	-	90°	Contacted both cusps simultaneously
Skupien	2016	Upper (1 root)	30	-	+	45°	Torward buccal cusp
Plotino	2017	Upper 1st	20	-	-	30°	Central fossa
Gurel	2016	Upper (1 root)	80	-	-	30°	Central fissure of the occlusal surface

**Table 2:** Heterogeneity summary, PDL Simulation, Load Inclination, Tip Position, Thermocycling in intact premolar subgroup.

Heterogeneity summary: **PDL simulation** (Binary; 0 = −PDL; +PDL) Number of studies: 17						
Group	df	Q	P > Q	Tau^2^	%I^2^	H^2^
0	11	741.34	0.000	91,125.915	98.52	67.39
1	4	1409.95	0.000	4.16e+05	99.72	352.49
Overall	16	396.53	0.000	5.38e+05	99.98	908.80
Test of group differences: Q_b = chi^2^ (1) = 0.75				Prob > Q_b = 0.386		
Heterogeneity summary: **Load inclination** (Binary; 0 = 30/45°; 1 = 90°) Number of studies: 17						
Group	df	Q	P > Q	Tau^2^	%I^2^	H^2^
0	7	2118.47	0.000	3.08e+05	99.67	302.64
1	8	243.53	0.000	52,820.161	96.71	30.44
Overall	16	14,540.83	0.000	5.38e+05	97.73	908.80
Test of group differences: Q_b = chi^2^ (1) = 2.77				Prob > Q_b = 0.096		
Heterogeneity summary: **Tip Position** (Binary; 0 = either buccal or lingual; 1 = central fossa or both cusps) Number of studies: 17						
Group	df	Q	P > Q	Tau^2^	%I^2^	H^2^
0	4	51.73	0.000	26,365.250	92.27	12.93
1	10	14,471.58	0.000	6.30e+05	99.93	1447.16
Overall	9	14,526.82	0.000	5.53+05	99.90	968.45
Test of group differences: Q_b = chi^2^ (1) = 0.58				Prob > Q_b = 0.447		
Heterogeneity summary: **Thermocycling** (Binary; 0 = −Therm; +Therm) Number of studies: 17						
Group	df	Q	P > Q	Tau^2^	%I^2^	H^2^
0	11	14,351.13	0.000	5.93e+05	73.46	3.77
1	4	88.66	0.000	58,364.924	98.98	97.81
Overall	16	14,540.83	0.000	5.38e+05	97.73	44.06
Test of group differences: Q_b = chi^2^ (1) = 0.09				Prob > Q_b = 0.766		

**Table 3:** Summarizing of results in subgroups with 0 walls lost.

Heterogeneity summary: **Load inclination** (Binary; 0 = 30/45°; 1 = 90°) Number of studies: 10						
Group	df	Q	P > Q	Tau^2^	%I^2^	H^2^
0	5	18.84	0.002	21,296.981	73.46	3.77
1	3	293.42	0.000	35,790.506	98.98	97.81
Overall	9	396.53	0.000	41,939.649	97.73	44.06
Test of group differences: Q_b = chi^2^ (1) = 8.97				Prob > Q_b = 0.003		

**Table 4:** Summarizing of load inclination and thermocycling subgroup results with 1 wall lost.

Heterogeneity summary: **Load inclination** (Binary; 0 = 30/45°; 1 = 90°) Number of studies: 6						
Group	df	Q	P > Q	Tau^2^	%I^2^	H^2^
0	0	0.00	-	0.000	-	-
1	4	378.35	0.000	84,712.462	98.94	94.59
Overall	5	378.41	0.000	74,292.555	98.68	75.68
Test of group differences: Q_b = chi^2^ (1) = 2.94				Prob > Q_b = 0.086		
Heterogeneity summary: **Thermocycling** (Binary; 0 = −Therm; +Therm) Number of studies: 6						
Group	Df	Q	P > Q	Tau^2^	%I^2^	H^2^
0	4	129.88	0.000	28,884.187	73.46	3.77
1	0	0.00	-	0.000	98.98	97.81
Overall	5	14,540.83	0.000	74,292.555	97.73	44.06
Test of group differences: Q_b = chi^2^ (1) = 50.83				Prob > Q_b = 0.000		

**Table 5:** Meta-regression of tip diameter in subgroup with 0 and 2 walls lost.

Meta es	Coef.	Std. Err.	z	P > z	[95% Conf.Interval]
Tip diameter	108.9722	109.2411	2.24	0.025	13.43596 204.5084
cons	92.22167	251.3299	0.37	0.714	−400.3759 584.8193
Test of group differences: Q_res = chi^2^ (13) = 396.23				Prob > Q_res = 0.0000	
Tip diameter	54.2533	21.42337	2.53	0.011	12.26426 96.24234
cons	272.2454	114.956	2.37	0.018	46.93586 497.555
Test of group differences: Q_res = chi2 (13) = 9033.13				Prob > Q_res = 0.0000	

## Supplementary Materials

The following are available online at https://www.mdpi.com/article/10.3390/dj9120146/s1, Figure S1: funnel plot for intact premolar group, Figure S2: funnel plot for 0 wall lost group; Figure S3: funnel plot for 1 wall lost group; Figure S4: funnel plot for 2 walls lost group.
