# Supplementary material for: Influence of Methodological Variables on Fracture Strength Tests Results of Premolars with Different Number of Residual Walls. A Systematic Review with Meta-Analysis
Source: Dent J (Basel). 2021 Dec 2;9(12):146. doi: 10.3390/dj9120146 (PMC8699883; doi:10.3390/dj9120146)
Supplement: Supplementary file 1 [file dentistry-09-00146-s001.zip › dentistry-1445977-supplementary.pdf]

Supplentary Materials

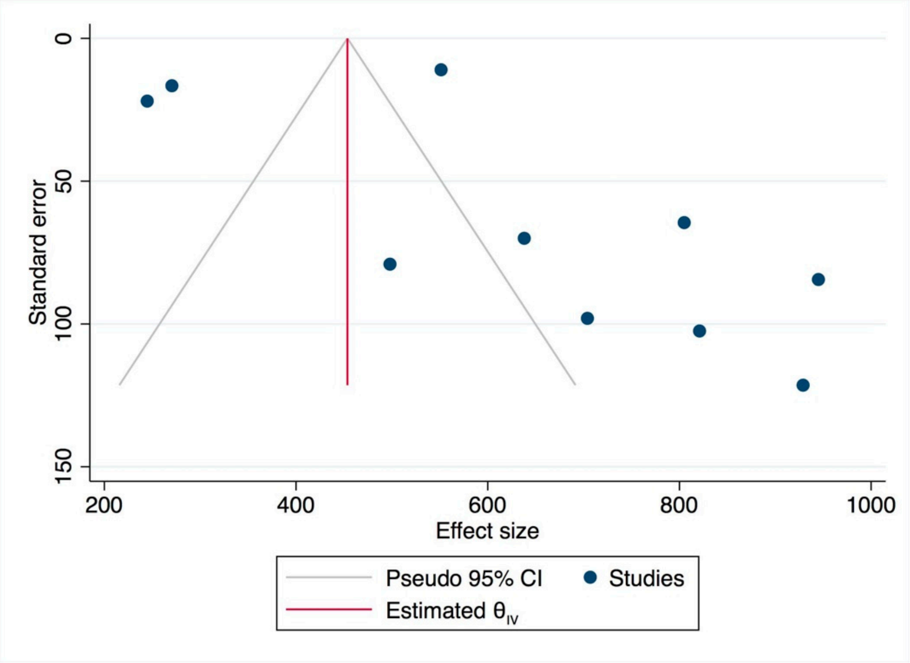

Figure S1: funnel plot for intact premolar group

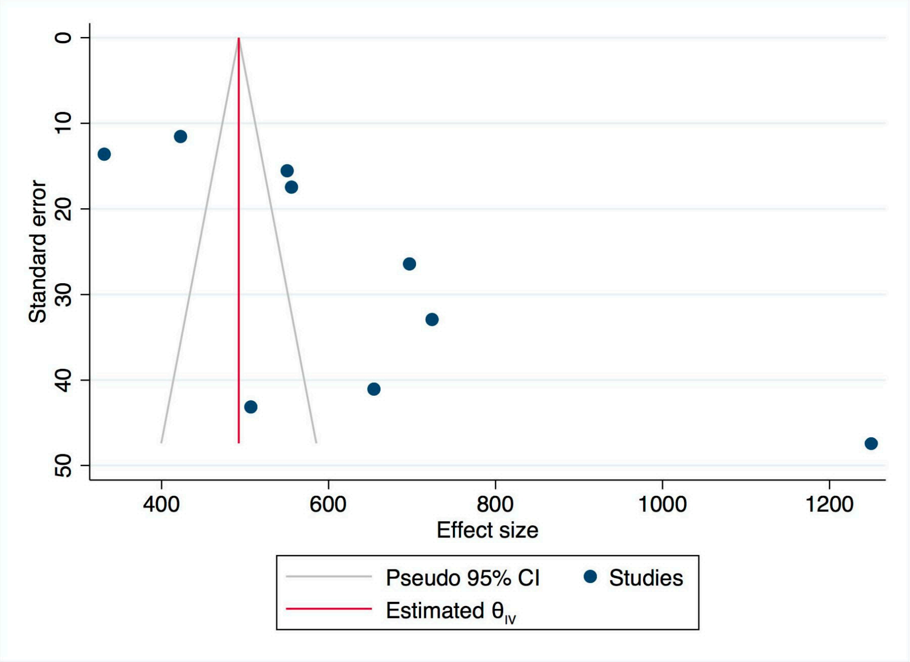

Figure S2: funnel plot for 0 wall lost group

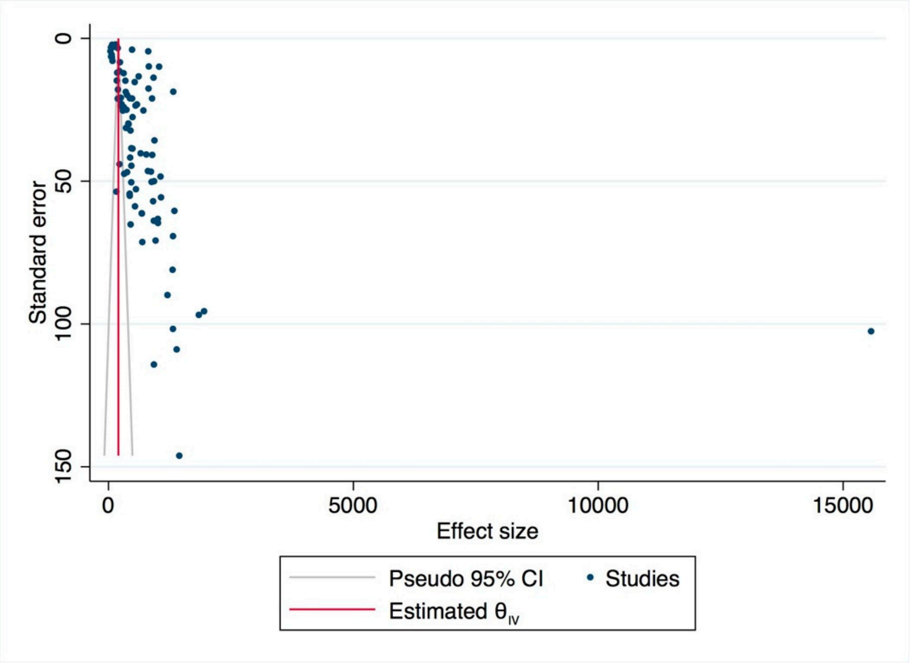

Figure S3: funnel plot for 1 wall lost group

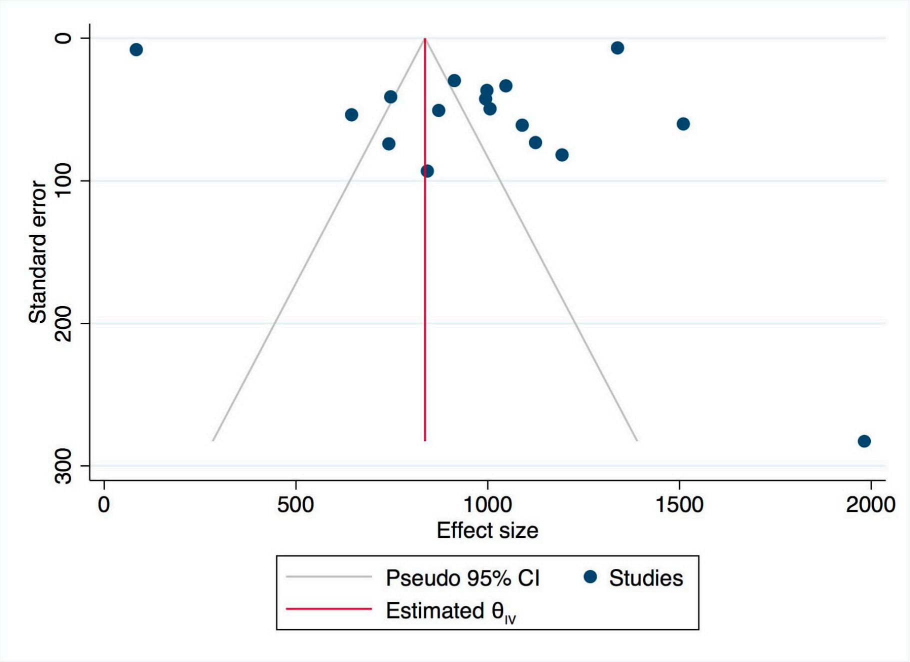

Figure S4: funnel plot for 2 walls lost group
